# Central New York Homœopathic Medical Society, September Meeting

**Published:** 1883-11

**Authors:** C. P. Jennings

**Affiliations:** Secretary


### THE SEPTEMBER MEETING OF THE CENTRAL NEW YORK HOMŒOPATHIC MEDICAL SOCIETY, IN SYRACUSE, N. Y., SEPTEMBER 20th, 1883.

The Society met at 10 A. M., in the office of Dr. Hawley, the President, A. E. Wallace, M. D., of Oneida, in the chair, fourteen members present, also one honorary member, E. Carleton, M. D., of New York city.

Drs. Schmitt, C. L. Swift (by Dr. Boyce), and Carleton read clinical papers, which were accepted and ordered to be published.

Dr. Harris read a paper on Contagion and Infection. Accepted and ordered to be published.

Dr. Boyce—Dr. Harris' paper brings out the dynamical in a clear way. I understand him to say that contagion is mechanical and infection dynamical. How can anything mechanical produce disease in the body, save in a dynamic way? The vital principle is not the spirit. They are different. Dr. Harris thinks they are one and the same. Is the vital principle the man? Is it your own proper self? Hahnemann thought that the disturbance of the vital principle results in disease. This disturbance may come from mechanical causes, and it may come from a man's spirit.

Dr. Schmitt—Dr. Harris says that contagion is mechanical and infection dynamical, and that disease by contagion is to be removed by mechanical means. I suppose he does not mean to convey the idea that syphilis, for example, should be treated by local applications, or the bite of a rabid dog by the cautery. We know these will fail.

Dr. Harris—I differ with some of my brethren as to the propriety of local applications. I use them sometimes. My paper says, where there is a mechanical cause at work, remove it by mechanical means, if necessary. If mechanical causes result in disturbance of the organism, treat the case dynamically.

Dr. Boyce—Does contagion act upon the physical nature or upon the spiritual? We are not conscious of what is going on within us. Contagion does not affect the vital principle except through the physical nature. There is something in us still, beyond and above the vital principle, viz.: the spirit. The vital principle prepares the body for the use of the spirit. This spirit is your own proper self. How does contagion or infection act upon the vital principle? Who can tell? To produce disease, the vital principle must be affected; to remove disease, the vital principle must be acted upon. But how? I have not been able to find out.

Dr. Harris—Probably we cannot know the how, nor all about the laws of spiritual life; but we may learn something about them, and should give our concentrated attention to them.

Dr. Nash.—Hahnemann made the distinction that it is impossible to know the causes of disease in their inner action, disease not being material, but dynamic. He taught that we must recognize this in order to cure the sick. If we know enough to cure disease, it is sufficient.

Dr. Wells—The great stumbling-block of the old school is their belief that diseases are material entities. Hence they fight disease with crude drugs in massive doses.

Dr. Schmitt—The old school go by fashions. Twenty-five years ago the liver was held responsible for a majority of diseases, and Calomel was their chief dependence. Now they attribute most diseases to malaria, in order that they may give Quinine. As to the connection between soul and body, I read Carpenter's work over and over, and could not understand him—do not think any one can.

Dr. Hawley—Am content to wait. Do not know much about it, and do not care to talk about it. One thing is certain, no man will ever be able to catch the causes of disease with the microscope.

Dr. Nash—Our material low-potency men want to see material in the potencies. They need to believe in the physiological action of drugs.

Dr. Gwynn—Where does the dynamic power of a drug reside? Is it wrapped up in a molecule? Why is it in drugs and not in something else?

Dr. Nash—Things which cannot be perceived by the senses we call spiritual. Investigations may show that the dynamical and the material always go together. The power is there in the substance, and you must attenuate the substance so that the physiological power may get hold of it and use it.

Dr. Schmitt—The newest theory is that attenuation separates the molecules. The molecule may have an exhalation. Attenuating a drug is supposed to set the molecules free to give forth their exhalations. We know that a man's foot gives forth, through the sole of his boot, a scent peculiar to itself, for his dog will detect the scent and will follow it alone among a thousand tracks of other men.

Dr. Hawley—As to material diseases spoken of awhile ago: Have a friend in New York city, an old-school physician, who thinks diabetes to be due to excess of sugar in the system. He is intent upon eliminating from flour all that can produce sugar, and he feeds his patients with what he calls diabetic bread.

Dr. Carleton—Allopathic advanced pathology now supposes the sugar in diabetic urine to be due to some lesion in the brain or in the spinal cord, not to the use of sugar in the food. As to contagion and infection, advanced pathologists of the old school are becoming chary of these terms. There is a difference between contagion and infection. Poisonous germs find their way into the human body. If they find a suitable soil there, they ferment and produce diseases. We have seen this proven in the Ward's Island Hospital, on the surgical side of it, in hot weather. Ulcers would become gangrenous, and progress so malignantly that amputation was necessary to save life. I recommended that a ward be prepared for the patients by using Chlorine gas as a disinfectant, then ventilating the ward. The wounds of the patients were washed with a solution of Permanganate of Potash, and the patients were removed into the prepared ward. The wounds went on in a healthy way. This would seem to prove that poison germs do produce disease. If, in a week or two, the patients were worse again, I would repeat the experiment.

Dr. Hawley, from the Committee on Necrology, reported the death of Dr. Schenck, and presented a minute for the action of the Society. The minute was adopted, and is as follows:

Benjamin Baird Schenck, M. D., was among the earliest converts from the old school of medicine to Homœopathy in the County of Onondaga, and he was always faithful to its law in his treatment of the sick. He was an organic member of this Society, and he was almost always present at and active in its meetings. He passed away from us on the 22d of March, 1883. In his death we, as a Society, feel that we have lost one of our best. He was true in all the relations of life; skillful, modest, and sympathetic as a physician; patriotic and public-spirited as a physician; noble, just, and true as a man—a Christian gentleman. As a Society, we extend our sympathies to his family and friends in their loss.

Lac caninum was selected for proving; proven to chose the potency.

Dr. Nash—If we use a fluxion potency we cannot know what potency we are proving.

Dr. Schmitt—It is desirable to have lady provers.

Drs. Harris, Hawley, and Seward were appointed a committee to report at the next meeting on provings, said committee to select drugs to be proved, to recommend the potencies to be used, and to instruct the provers as to the plans of proving.

Dr. Hawley—Had another Cina case a few days ago. Was called to a child ten years old. The parents thought she was going into convulsions. She has been, for four weeks, suffering from sudden attacks, in which she lost the control of her voice and spoke like a person without a palate. Had difficult breathing; must be fanned. Could not bear to have the nose touched, yet, when the fingers were placed firmly on the sides of the nose, she would be quiet. An old-school physician had been treating her. He pronounced the case catarrhal. Throat in good condition. At times marked pallor about the mouth. Temperament nervous. She was subject to attacks in which she would complain of lameness of a finger, or of an arm, or of a knee. Clearly it was a case of reflex action of intestinal irritation. Gave Cina$^{200}$. The next day she was up and about the house, well and bright.

Dr. Nash—Did you prescribe upon your pathological idea of the case?

Dr. Hawley—No; never do.

Drs. Schmitt, Nash, and Wells were appointed essayists for the next meeting, they to choose subjects.

Dr. Wells was requested to give the history of Homœopathy in the State of New York.

Lycopodium was selected for conversation.

Dr. Harris—Came of a catarrhal family. Suffered much from catarrh while living in New York, before going West. On one occasion took Lycopodium$^{30}$. It put me to bed. Head and eyes had to be covered up. Head ached as if it would split open. After that I was free from catarrhal attacks, and still continue to be better. Living in Wisconsin may have helped the improvement.

Dr. Young—The characteristic sediment in the urine has led me to give Lycopodium, and with success.

Dr. C. L. Swift—In diphtheria, where Belladonna seemed to be indicated and failed, the throat being sore on the right side, Lycopodium relieved. Other Lycopodium symptoms were present, as aggravation toward night; warm drinks relieved.

Dr. Carleton—Its characteristics led me to give it, and with success. One of these is aphasia; another is entire prostration of body and mind. Am always in doubt how to obtain its good effects without aggravation; whether to give the 30th, or the 200th, or millionth. The CM has aggravated badly. Am always sorry when I find it indicated.

Dr. Besemer—Have used it successfully in catarrhal cases, dry catarrh; nose stopped; patient breathes with his mouth open; gas in the bowels; the characteristic sediment in the urine; 4 P. M. aggravation, especially when the patient is fond of sweet things.

Dr. Wells—A woman has, for six years past, been compelled to use cathartics or injections in order to procure a movement of the bowels at all. Abdomen badly bloated. Aggravation about 4 P. M. Has taken a good deal of Nux vomica. Three days ago she applied to me. Gave Lycopodium, 30th trit. In forty-eight hours she had a free movement. Fæces a long string, coiled up, partly flattened.

Dr. Schmitt—Have found Lycopodium useful in labor cases. The key-note is: She cannot keep still in the pains, wants to walk or move about during the pains. Lycopodium overcomes undilutable os.

Have had experience with it for myself. Had swallowed accidentally a piece of a tooth-pick. Was threatened with peritonitis; could lie upon my right side only; was restless. While hesitating what to take, noticed that my tongue took on a pendulous motion. This suggested Lycopodium. Took it. Relief was prompt.

Once my horse was sick with a heavy cold. Found her lying flat. Respiration about twenty-four. She was bloated. Her nostrils were flapping with a wing-like motion of the alæ. This last symptom suggested Lycopodium. Gave it at 10 P. M.; at 11 P. M. she was well, was up, and eating heartily.

Dr. Nash—In soreness of the right side of the throat, if there be stuffing of the nose, Lycopodium acts quickly and successfully, but it fails if the nasal symptom be absent. Have relieved many cases of chronic liver affection with Lycopodium when there were present these symptoms: Flatulence; sense of hunger, soon satisfied; even satiety when beginning to eat. Used Jenichen's 6M.

A woman had continual desire for stool, strangury, red sand in urine. Cured by one dose of Lycopodium, although it aggravated her condition for two days. Have verified the 4 P. M. aggravation many times.

Dr. Jennings—Was waiting upon a case of malignant diphtheria. The evening of the fifth day, felt suddenly a peculiar tinging at tip of tongue. It propagated itself along the right half of the tongue, and in a few minutes extended to right side of the throat. Took a grain of Lycopodium, 30th trit. In less than half an hour the sensation disappeared. In the course of the next two weeks the sensation returned twice in the same region as at first. Both times Lycopodium, 30th trit., extinguished it.

Dr. Schmitt—Hering says that in chronic diseases it is better to give before Lycopodium another not anti-psoric medicine. How is this? Have seen Lycopodium relieve chronic constipation quickly. On the return of the trouble Lycopodium had no effect.

Dr. Jennings—Lippe has the same remark. Perhaps, in chronic cases, where Lycopodium proves ineffectual when repeated, though successful at first, the failure is due to giving Lycopodium without preceding it by another not antipsoric medicine.

The Secretary was surprised with a handsome testimonial presented to him by Dr. Seward in behalf of the members of the Society. He expressed his thankful sense of the kindness.

Adjourned.

C. P. Jennings, *Secretary.*

